# Assessing the impact on caregivers caring for patients with rare pediatric lysosomal storage diseases: development of the Caregiver Impact Questionnaire

**DOI:** 10.1186/s41687-019-0140-3

**Published:** 2019-07-23

**Authors:** Magdalena Harrington, Asha Hareendran, Anne Skalicky, Hilary Wilson, Marci Clark, Jaromir Mikl

**Affiliations:** 1 Shire (a member of the Takeda group of companies), Lexington, MA USA; 2 Present Address: Pfizer, 610 Main St, Cambridge, MA 02139 USA; 3 Evidera, Bethesda, MD USA; 4 Present Address: Boehringer Ingelheim Pharmaceuticals, Inc., Ridgefield, CT USA; 5 RTI Health Solutions, Ann Arbor, MI USA; 6 Present Address: Purdue Pharma, Stamford, CT USA

**Keywords:** Caregiver questionnaire, Lysosomal storage disease, Metachromatic leukodystrophy, MLD, MPS II, MPS IIIA, Mucopolysaccharidosis type II, Mucopolysaccharidosis type IIIA

## Abstract

**Background:**

Capturing the impact of caring for patients with debilitating rare disease is important for understanding disease burden. We aimed to develop and validate an instrument to measure the impact on caregivers of caring for children with three lysosomal storage diseases (LSDs): metachromatic leukodystrophy (MLD), neuronopathic mucopolysaccharidosis type II (MPS II) and mucopolysaccharidosis type IIIA (MPS IIIA).

**Methods:**

A draft instrument was developed based on targeted literature searches and revised through sequential qualitative interviews with caregivers of patients first with MLD (*n* = 16), then with MPS II (*n* = 22), and finally with MPS IIIA (*n* = 8). The instrument, which covered domains of physical, emotional, social and economic burden, was refined at each stage of development based on caregiver feedback. Saturation of major concepts was reached during concept elicitation (MLD and MPS II).

**Results:**

It was confirmed that caring for a patient with an LSD impacts social functioning, emotional/psychological functioning, physical functioning, daily activities, and finances/work productivity. Results from cognitive debriefing of the draft questionnaires were considered during each round of interviews, resulting in a final set of items that caregivers found clear and easy to understand. The Caregiver Impact Questionnaire (CIQ) has 30 items in five domains: (1) social functioning (7 items); (2) impact on daily activities (5 items); (3) emotional/psychological functioning (10 items); (4) physical functioning (6 items); and (5) financial impact (2 items).

**Conclusions:**

These findings demonstrate that the content of the CIQ is relevant for determining the impact of caring on caregivers of patients with MLD, MPS II and MPS IIIA.

**Electronic supplementary material:**

The online version of this article (10.1186/s41687-019-0140-3) contains supplementary material, which is available to authorized users.

## Background

Debilitating diseases affecting children can place an enormous physical, emotional, social, and financial burden on the people providing long-term, continuous care for affected patients. The inherited metabolic diseases metachromatic leukodystrophy (MLD), mucopolysaccharidosis type II (MPS II, also known as Hunter syndrome) and mucopolysaccharidosis type IIIA (MPS IIIA, also known as Sanfilippo A syndrome) result from decreased production of one or more of the lysosomal hydrolases [1]. These rare lysosomal storage diseases (LSDs) lead to severe life-limiting effects, for which patients can require intensive 24-h care (Table 1).

**Table 1:** Summary of general disease characteristics of MLD, MPS II and MPS IIIA

Disease	Types	Age of onset	Life expectancy	Primary symptoms	Key similarities/differences within disease types	Similarities between MLD, MPS II and MPS IIIA
MLD	Late-infantile (onset before 3 years of age) [2]	Median 1.5 years [3]	Mean age at death 4.2 years [4]	Motor related (e.g. weakness, gait abnormalities, quadriparesis, dysarthria, hearing difficulties, vision impairment, incontinence) [5].	Motor decline is typical for both late-infantile and juvenile MLD (more rapid in late-infantile); in the juvenile form, it may be preceded by cognitive and behavioral problems [2].	All three diseases tend to have a pediatric onset and are associated with significantly reduced life expectancy. Juvenile MLD, severe MPS II and MPS IIIA are associated with behavioral problems and eventual motor decline. In late-infantile MLD, patients develop motor deficits very young, making manifestations of behavioral problems difficult to detect.
	Juvenile (onset before 16 years of age) [2]	Median 6 years [3]	Mean age at death 17.4 years [4]	Neuropsychiatric or cognitive prodrome (i.e. frontal lobe dysregulation, followed by gradual neurologic decline) [6].		
MPS II	Severe (neuronopathic) – two-thirds of patients, with signs and symptoms appearing by 3 years of age [7]	Median 1.5 years [8]	Median age at death 11.7 years [9]	Affects multiple organs and physiologic systems (e.g. facial dysmorphism, organomegaly, joint stiffness and contractures, pulmonary dysfunction, myocardial enlargement and valvular dysfunction, and neurologic involvement). In patients with neurologic involvement, intelligence is impaired [10].	Patients with the severe form of MPS II have cognitive impairment; patients with the less severe form do not experience significant cognitive involvement [9].	
	Mild (non-neuronopathic)		Median age at death 14.1 years [9]	Individuals with non-neuronopathic MPS II are of normal intelligence [11].		
MPS IIIA	NA	Mean 3 years [12]	Median age at death 15.0 years [12]	Primarily characterized by degeneration of the central nervous system, resulting in severe cognitive impairment (e.g. speech delay) as well as hyperactivity and aggressive behavioral problems [13]. Behavioral difficulties tend to become increasingly severe for 5 or 10 years, after which there is a regression in behavioral disturbances, which is associated with a progressive and severe loss of intellectual and motor functioning [13]. Somatic symptoms include coarse facial features and skeletal pathology that affects growth and causes degenerative joint disease, hepatosplenomegaly, macrocrania and hearing loss [13].	The clinical spectrum in MPS IIIA is broad (e.g. patients typically survive until early teens in the most severe cases or as late as the sixth decade in attenuated forms) [13].	

*MLD* metachromatic leukodystrophy, *MPS II* mucopolysaccharidosis type II, *MPS IIIA* mucopolysaccharidosis type IIIA, *NA* not applicable

The impacts of these diseases extend beyond the patients, dramatically altering the lives of their families and caregivers [14]. Collecting information on caregiver burden would help with documenting the impact of disease on caregivers and monitoring their well-being. This would, in turn, help identify the need for support services and evaluate outcomes of interventions, including the value to caregivers of treatments that delay or prevent disease progression. Tools that collect data about disease-specific experiences are better suited to identify specific needs and likely to be more sensitive to changes resulting from interventions.

Here, we aimed to develop an instrument that captures the impact of caring among caregivers of children with MLD, MPS II and/or MPS IIIA, and to assess the content validity of this instrument.

## Methods

**Fig. 1:**
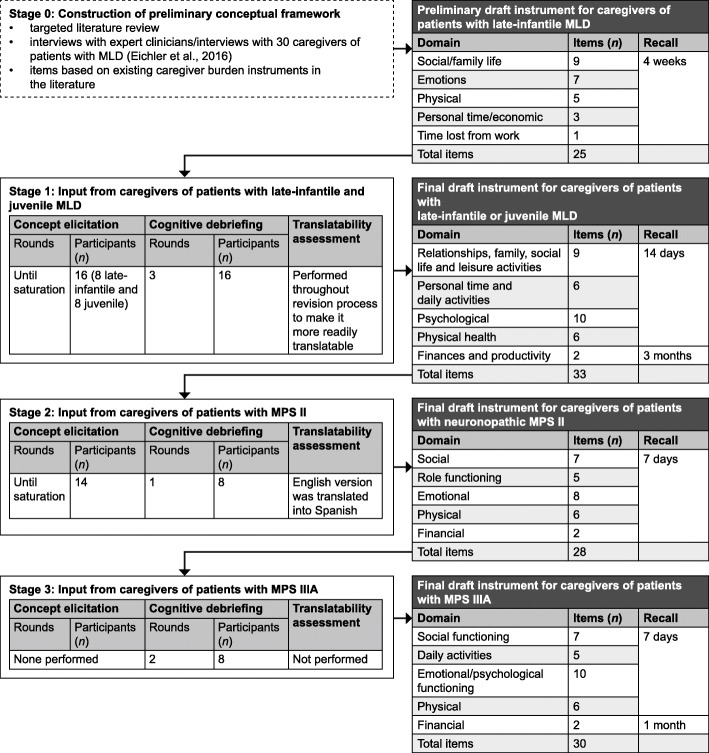
Study design. *MLD*, metachromatic leukodystrophy; *MPS II*, mucopolysaccharidosis type II; *MPS IIIA*, mucopolysaccharidosis type IIIA

During stage 0, a targeted literature review was performed to identify relevant concepts and caregiver burden instruments. Based on these data and interviews conducted with health professionals and caregivers of patients with MLD [14], a preliminary instrument was developed for further testing in three stages (stages 1–3) of review by caregivers, resulting in revision of the instrument (Fig. 1). Stage 1 of the Caregiver Impact Questionnaire (CIQ) development involved interviews with caregivers of patients with MLD; stages 2 and 3 involved interviews with caregivers of patients with MPS II and MPS IIIA, respectively. For stages 1 and 2, data saturation of the concepts was required to justify item retention or addition. This method ensures that sufficient data are collected to support the findings: when no new information emerges in successive interviews, saturation has been reached [15].

Stages 1–3 of CIQ development were performed by researchers representing several companies, who were experienced in measurement development, and by experienced qualitative interviewers in conjunction with a sponsor drug development team (Shire, Lexington, MA, USA; a Takeda company). Stage 0 was performed by Evidera (Bethesda, MD, USA) and the sponsor. Stages 1 and 2 were conducted by Evidera and stage 3 by RTI Health Solutions (Ann Arbor, MI, USA).

### Stage 0: development of the preliminary draft MLD-CIQ

**Fig. 2:**
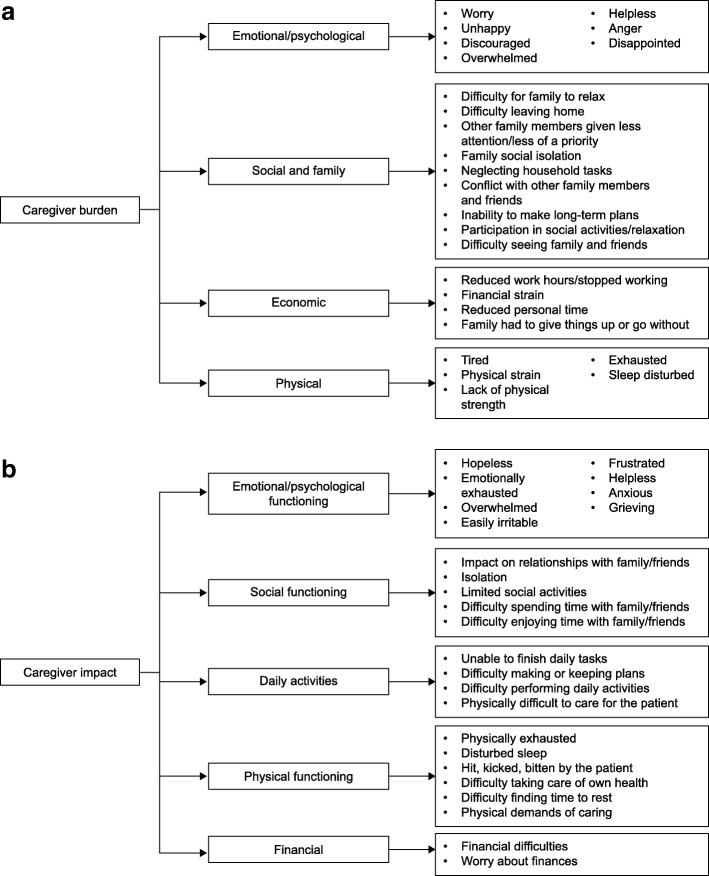
**a** Preliminary conceptual framework. **b** Revised conceptual framework after completion of all patient interviews

A preliminary conceptual framework (Fig. 2a) was generated based on a literature review of 53 publications related to MLD, and interviews were conducted with expert clinicians and caregivers of patients with MLD [14]. In addition, a targeted literature search for existing caregiver burden instruments was performed to help inform item development. The search was pragmatic in nature, and used the MEDLINE database (National Library of Medicine, Bethesda, MD, USA) and the Patient-Reported Outcome and Quality of Life Instruments Database (Mapi Research Trust, Lyon, France) to identify existing instruments that measure caregiver strain and burden.

The 23 instruments identified were relatively diverse, having been developed for use with caregivers across a range of different conditions, including psychiatric illnesses, chronic physical impairment and diabetes. Overall, they covered a broad range of concepts relating to emotional, physical, social and economic impacts of caring. However, many had been generated before the availability of the Food and Drug Administration instrument development guidelines [16], and very few were developed with direct input from caregivers of pediatric patients. In addition, none had been specifically developed for caregivers of children with the motor and cognitive dysfunction associated with LSDs. As these existing instruments would require substantial revision to adequately address caregiver impact in the context of LSDs, the decision was made to proceed with the development of a new instrument. Items for the draft MLD-CIQ were developed within the conceptual framework (Fig. 2a), based on concepts identified from the literature review and interviews with clinicians and caregivers [14] and items identified in the existing caregiver burden instruments.

An outline of the preliminary draft MLD-CIQ is provided in Fig. 1.

### Stages 1–3: testing and refining the instrument

#### Participants

Potential participants were initially approached by the MLD Foundation, the International MPS Network (in the USA, UK, Spain and Mexico) or the National MPS Society (in the USA) via email or printed invitations, and were recruited consecutively based on eligibility. To be included in the MLD and MPS II interviews, caregivers had to be at least 18 years old. In addition, criteria were imposed to limit the caregiver population to those who were caring for patients with the most severe and earliest presenting forms of these diseases, in order to capture the full extent of potential impacts on caregivers. For MLD, only caregivers of patients with late-infantile MLD (defined as symptom onset between 6 months and 4 years of age inclusive) or juvenile MLD (symptom onset at 5–18 years of age) were eligible. For MPS II, only caregivers of patients aged 3–18 years with a diagnosis of neuronopathic MPS II were eligible. For MPS IIIA, only primary caregivers of patients aged 1–10 years were eligible and, although no caregiver age requirement was specified, they were required to be a parent or family caregiver (i.e. not paid). For all three diseases, the patient’s physician-confirmed diagnosis was self-reported by the caregiver. In addition, for MLD and MPS II, patient stakeholders were engaged in the instrument development process. For MLD, these stakeholders were two patient caregiver advocates, who were identified through the MLD Foundation and reviewed the CIQ. For MPS II, the patient stakeholder was a caregiver consultant in MPS II, identified through the MPS Society, who reviewed the study protocol and interview guides.

#### Interview process

All study materials were approved by appropriate ethics committees. All interviews in the UK, USA and Canada were conducted in English, and five interviews with caregivers of patients with MPS II were conducted in Spanish (Spain and Mexico). The English versions of the CIQ and study documents were translated into Spanish by professional patient-reported outcome translators at FACITtrans (http://www.facit.org/TransHome) according to established standards [17].

Study documents were mailed to participants before the telephone interview, except for one MPS IIIA interview, which was conducted in person. Participants were told not to open the documents until asked to by the interviewer, to ensure that the instructions and purpose of the study could be fully explained first, and to maintain spontaneity in responses to questions. Interviews were audio-recorded with the caregivers’ permission and verbatim transcripts were produced. Before each interview, the interviewer introduced themselves as a researcher for a company conducting the study on behalf of Shire (a Takeda company), and explained the study aims and procedures in detail. Consent was obtained either during interview scheduling or immediately before the interview, once study eligibility had been confirmed. For telephone interviews, the interviewer discussed the consent form with participants and asked them to confirm their consent verbally. For the face-to-face interview, written consent was obtained in person before the interview.

Except for one face-to-face MPS IIIA interview, all interviews were conducted by telephone; it was recommended that participants find a quiet place for the call away from distractions. The interviewers followed a semi-structured interview guide, which included concept elicitation [18, 19] for MLD and MPS II and cognitive debriefing [20, 21] of the CIQ completed for all three diseases (see Additional file 1 for a summary of key themes of the interview guides). The cognitive debriefing conducted with MPS IIIA caregivers was performed to identify whether the items were relevant for this condition and understood by caregivers, and participants were also given an opportunity to describe any relevant concepts that were not already covered.

Each interview lasted approximately 90 min for MLD, and the caregiver-completed CIQs were returned via post to the study center. For MPS II, concept elicitation lasted 60–90 min and cognitive debriefing lasted 90–120 min. For MPS IIIA, cognitive debriefing lasted approximately 60 min.

#### Concept elicitation

Concept elicitation interviews [18, 19] began with a conversation about the caregiver’s experiences of taking care of the child with MLD or MPS II to elicit a description of the impacts of caregiving on their life. Caregivers were asked to describe specifically how caring for someone with MLD or MPS II impacts their relationships, family, social life, leisure activities, personal time, daily activities, physical and mental health, work productivity, and finances. Caregivers were probed on their experiences with caregiving at the domain level (e.g. how does providing care for your child affect your other family relationships?). The concepts elicited from the interviews were compared with the concepts in the draft CIQ instrument.

A saturation grid was used to capture the caregiver burden concepts experienced by participants, to identify if further interviews were required.

#### Cognitive debriefing

During cognitive debriefing [20, 21], the caregivers’ understanding of the instrument and the appropriateness of recall periods and response options were tested. Caregivers were asked about: (1) the questionnaire’s instructions; (2) the content coverage of the questionnaire to ensure items fully captured relevant concepts; (3) the clarity of the items within the scale; (4) how the participants interpreted the items; (5) ease of completion of the scale; (6) comprehensiveness and relevance of the measure; (7) the appropriateness of the format, response scales, and recall period. Participants engaged in a standard ‘think-aloud’ cognitive interview [20, 22], reading the instructions and questions aloud and verbalizing their thought process as they answered the questions and described the meaning of the questions in relation to their caregiver experience.

For MLD, the cognitive debriefing interviews were performed in three rounds, with changes made to the questionnaire after each round and the interview guide modified accordingly. During the revision process, a translatability and reading-level assessment was conducted to make the questionnaire more readily translatable into other languages. One round of cognitive debriefing was performed for MPS II. Two rounds of cognitive debriefing were performed for MPS IIIA, with revision of the instrument between rounds. A sociodemographic questionnaire was also completed by all caregivers.

### Analysis

In stages 1 and 2 of CIQ development with MLD and MPS II caregivers, data from concept elicitation and cognitive debriefing interviews were collected and the wording revised until comprehension of items was reached. The resulting questionnaire was tested in stage 3. For stages 1 and 2, content analysis of the concept elicitation and cognitive debriefing interview data was performed using MS Excel, including documentation of saturation. For stage 3, notes from the first round of cognitive interviews were analyzed, and revisions to the draft CIQ were made based on caregiver feedback. After the last round of cognitive interviews in MPS IIIA, analysis was conducted by detailed evaluation of transcripts and interview notes to inform the final set of CIQ items, and a revised conceptual framework was generated (Fig. 2b).

## Results

### Sample characteristics

In stage 1, 16 caregivers for patients with MLD were interviewed (six in each of the first two rounds of interviews, four in the final round). Eight were caring for patients with late-infantile MLD and eight for patients with juvenile MLD. Twenty-two caregivers for patients with MPS II were interviewed in stage 2 (14 in concept elicitation, eight in cognitive debriefing), and eight caregivers for patients with MPS IIIA in stage 3 (four in each of two rounds of cognitive debriefing). The mean age of caregivers was late 30s to early 40s, over 85% were female, and for MLD and MPS II at least 82% were married and the mother of the child (parental/marital status was not reported for MPS IIIA; Table 2). Over 50% of caregivers were employed and most (74%) had received a college or university education (Table 2). The mean age (range) of index patients with late-infantile MLD, juvenile MLD, MPS II and MPS IIIA was 4.7 years (3–11), 19.6 years (9–28), 8.5 years (3–15) and 6.9 years (3.5–10), respectively.

**Table 2:** Caregiver demographics

Characteristic	MLD (CE, CD) *N* = 16	MPS II (CE) *N* = 14	MPS II (CD) *N* = 8	MPS IIIA (CD) *N* = 8
Age (mean), years	43.3 (SD 9.5)	37.9 (SD 6.3)	41.3 (SD 6.9)	38.1 (range 30–48)
Sex, n (%)				
Male	1 (6.2)	2 (14.3)	0	1 (12.5)
Female	15 (93.8)	12 (85.7)	8 (100.0)	7 (87.5)
Ethnic background, n (%)				
Hispanic or Latino	1 (6.2)	NR	NR	NR
Non-Hispanic and non-Latino	15 (93.8)	9 (64.3)	4 (50.0)	NR
Not asked/reported	0	5 (35.7)^a^	4 (50.0)^a^	8 (100.0)
Racial background, n (%)				
Black	0	1 (7.1)	0	0
White	15 (93.8)	8 (57.1)	3 (37.5)	8 (100.0)
Asian	1 (6.2)	NR	0	0
Mixed African/White	0	NR	1 (12.5)	0
Not asked/reported	0	5 (35.7)^a^	4 (50.0)^a^	0
Country of residence, n (%)				
USA	13 (81.3)	7 (50.0)	2 (25.0)	8 (100.0)
Canada	2 (12.5)	0	0	0
UK	1 (6.2)	2 (14.3)	2 (25.0)	0
Mexico	0	3 (21.4)	2 (25.0)	0
Spain	0	2 (14.3)	2 (25.0)	0
Relationship to child, n (%)				
Mother	15 (93.8)	12 (85.7)	8 (100.0)	NR
Father	1 (6.2)	2 (14.3)	0	NR
Living status (for MPS II), n (%)				
Married (living with partner, family or friends)	14 (87.5)	12 (85.7)	8 (100.0)	NR
Single (living alone)	2 (12.5)	1 (7.1)	0	NR
Other living arrangement, e.g. widowed	0	1 (7.1)	0	NR
Employment status, n (%)				
Employed full/part time	8 (50.0)	9 (64.3)	4 (50.0)	5 (62.5)
Homemaker	7 (43.8)	5 (35.7)	4 (50.0)	NR
Unemployed/retired	1 (6.2)	0	0	3 (37.5)
Highest education level, n (%)				
Elementary/primary school	0	2 (14.3)	0	0
High/secondary school	2 (12.5)	3 (21.4)	1 (12.5)	0
Associates degree, vocational, technical or trade school	0	1 (7.1)	3 (37.5)	0
Some college	3 (18.8)	1 (7.1)	3 (37.5)	3 (37.5)
College/university degree	9 (56.3)	5 (35.7)	0	2 (25.0)
Postgraduate/advanced/professional degree	2 (12.5)	2 (14.3)	1 (12.5)	3 (37.5)

*CD* cognitive debriefing, *CE* concept elicitation, *MLD* metachromatic leukodystrophy, *MPS II* mucopolysaccharidosis type II, *MPS IIIA* mucopolysaccharidosis type IIIA, *NR* not reported, *SD* standard deviation

^a^Not asked for caregivers in Mexico and Spain

### Overview of interview findings

Table 3 provides an overview of the major changes made to the CIQ in response to input from caregivers, and the results are detailed sequentially below. Table 4 shows the overall domains of the CIQ versions, alongside illustrative descriptions of caregiver impacts based on quotes obtained during interviews. Direct quotes were not reported in order to protect caregivers’ identity, given the extreme rarity of these diseases.

**Table 3:** Summary of major changes made to the CIQ

Type of change	Preliminary MLD-CIQ (25 items) Concepts present in preliminary draft	MLD-CIQ final draft (33 items) Concepts added or removed		MPS II-CIQ (28 items) Concepts added or removed		MPS IIIA-CIQ (30 items) Concepts added or removed	
		*Revision*	*Rationale*	*Revision*	*Rationale*	*Revision*	*Rationale*
Social/family life/leisure/relationships	Difficulty in: relaxing; participating in social activities; leaving home; completing household tasks; seeing family and friends; making long-term plans; paying attention to other family members or causing conflict between family members	Concepts added: Effort to go out in public with patient	Descriptions of difficulty taking child out of the house, not related to social stigma	Concepts added: Difficulty due to the patient’s disruptive behavior Effects of disruptive behavior on: (1) enjoying time with family or friends; (2) doing daily activities; (3) caring for the patient; (4) social activities; (5) managing the patient’s behavior	Overarching concept raised by caregivers, reflecting the behavioral symptoms common in severe MPS II	No new concepts added or removed	
		Effort to participate in leisure activities	Descriptions by caregivers of leisure activities being impacted				
		Effort to communicate with patient	Suggestion to focus on the frequency of communication difficulties, because not all patients are unable to communicate				
Emotional/psychological functioning	Worried; unhappy; overwhelmed; helpless; angry; discouraged; disappointed that you cannot communicate with your child	Concepts added: Easily impatient or irritable; discouraged about limited treatment options; feel stressed	Frequent reports of general stress and specific stressful events	Concepts added: Frustrated that you could not communicate with the patient	Raised by caregivers as an important concept not covered by existing questions Removed as an approved treatment is available for MPS II Deemed not specific enough	Concepts added: Anxious about the patient’s future; grieving about the patient’s illness; being hit, kicked, or bitten by the child	All raised by caregivers as important concepts that were not covered by existing questions
				Concepts removed: Discouraged about limited treatment options Worried			
Physical functioning	Tired; physical strain; do not have enough physical strength to care for your child; feel exhausted; sleep disturbance	Concepts added: Unable to take care of own health	Reports of delayed caregiver treatment or neglect of own health due to prioritizing caregiving	Concepts added: Physically exhausted		Concepts removed Difficulty dealing with disruptive behavior	Deemed redundant (covered by other questions)
Daily activities	Families giving up things they usually do; personal time reduced	Concepts added: Unable to participate in activities because of limited time	Range of activities ‘given up’ by caregivers (e.g. seeing family, exercising)	No new concepts added or removed		No new concepts added or removed	
Financial/productivity	Financial strain on family; days missed from work	Concepts added: Can’t participate in activities because of limited finances Concepts removed: Days missed from work	Focus shifted to the effect of financial strain on the caregiver rather than the whole family	Concepts added: Worry about finances Concepts removed: Can’t participate in activities because of limited finances	Endorsed concepts of ‘worry’ and ‘financial difficulties’	No new concepts added or removed	

*CIQ* Caregiver Impact Questionnaire, *MLD* metachromatic leukodystrophy, *MPS II* mucopolysaccharidosis type II, *MPS IIIA* mucopolysaccharidosis type IIIA

**Table 4:** Examples of specific impacts described by caregivers, which demonstrate important concepts and modifications of each version of the CIQ

Preliminary MLD-CIQ (25 items)	MLD-CIQ final draft (33 items and general QOL)	MPS II Hunter-CIQ (28 items)	MPS IIIA-CIQ (30 items)
Impact on social and family life	*Impact on relationships, family, social life, and leisure activities* Inability to participate in social activities because child requires constant care. Family members need to visit the caregiver’s house because the caregiver is unable to leave the house. Feeling reluctant to go out due to caring responsibility for the child.	*Social functioning* *Your relationships with family members or friends* Not able to spend as much time with spouse. Child’s behavior negatively impacts relationships between parents. Child’s behavior puts a strain on marital/romantic relationship and limits opportunities to go out as a couple. *Did you feel isolated from other people?* Feeling of isolation due to limited social interactions. Social interactions are limited to in-home nurses or therapists. Feeling of being confined to home because child does not cope well with being outside.	*Social functioning* *Your relationships with family members or friends* Difficulty participating in family activities with the child due to their behavior; often need to cut family visits short. Inability to leave home. Social interactions limited to phone calls. *Feeling isolated* Finding it difficult to visit family. Feeling isolated due to being unable to go out easily with the child.
Impact on emotions	*Psychological impact* Feeling helpless due to inability to cure the child. Losing temper easily over small things due to strain of caring for the child. Inability to understand and communicate with the child.	*Emotional/psychological functioning* *Did you feel sad (changed to emotionally exhausted) about the patient’s illness?* Feeling bothered, depressed, crying a lot. Having depression as a result of seeing the child’s disease progressing.	*Emotional/psychological functioning* *Being hit, kicked, or bitten* Being frequently kicked, hit, pinched, bitten or slapped by the child. Feeling that the child lashing out indicates anger or frustration towards family members. *Feeling anxious about the patient’s future* Thinking about the child dying. Feeling anxiety and fear about the future. *Grieving about the patient’s illness* Feeling hopeless, frustrated, helpless, and emotional. Regularly recurring feeling of grief.
Physical impact	*Impact on physical health* Feeling exhausted. Unable to fall asleep due to extreme exhaustion. Difficulty in picking the child up and moving them from place to place. Unable to physically lift the child.	*Physical functioning* *Did you feel tired (changed to physically exhausted) as a result of taking care of the patient?* Feeling exhausted and drained; needing to nap.	*Physical functioning* *Difficulty dealing with disruptive behavior* Caring for the child and dealing with disruptive behavior is considered as part of taking care of the child. Disruptive behavior impacts emotional and physical functioning.
			*How often did you feel physically exhausted as a result of taking care of the patient?* Feeling worn out due to the child’s hyperactivity. Feeling tired due to the ongoing need to care for the child and assist with activities of daily living. Feeling physically exhausted due to emotional burden.
Impact on personal time	*Impact on personal time and daily activities*	*Role functioning*	*Impact on daily activities*
	Not enough time to do any activities other than caring for the child. No time to relax due to constant care requirements.	*How difficult was it for you to do your daily activities at work or home because you needed to take care of the patient? (Changed to how difficult was it for you to do your daily activities at home or at work because you needed to take care of the patient?)* Inability to leave child unattended impacts the ability to do daily chores. All aspects of daily life are impacted as a result of caring for a severely disabled child.	*How difficult was it for you to do your daily activities at work or home because you needed to take care of the patient?* Inability to let the child out of sight makes it almost impossible to do daily chores. Feeling unable to continue working. Work is impacted by management of the child’s medical care (e.g. scheduling medical appointments)
Economic impact	*Impact on finances and productivity*	*Financial impact*	*Financial impact*
	Inability to afford discretionary expenses, e.g. leisure travel. Financial impact due to inability to work. Living on a single income. Work productivity is negatively impacted. Living paycheck to paycheck.	*Significant costs associated with care and home modifications including paying for:* “pull-ups”, “babysitter”, “a special stroller”, “a bike for special needs children”, “nebulizer”, “expanded bathtub”, “ramp”, “removing carpet”, “special van with wheelchair access”, “gas and hotel for doctor visits”.	*How often did providing care for the patient cause you financial difficulties?* Constant financial impact from the costs of medical care and tests. Financial difficulties due to out-of-pocket expenses and lack of insurance reimbursement for medical supplements.
		*Significant impacts on work productivity* Work gets interrupted to care for/attend the child. Need to take time off from work to care for the child. Changing jobs frequently as a result.	Financial necessity for both parents to work due to costs associated with medical care.

The descriptions given are based on direct quotes obtained from caregivers during the interview process

*CIQ* Caregiver Impact Questionnaire, *MLD* metachromatic leukodystrophy, *MPS II* mucopolysaccharidosis type II, *MPS IIIA* mucopolysaccharidosis type IIIA, *QOL* quality of life

#### Stage 1: concept elicitation in MLD

Interviews with caregivers of patients with MLD were conducted using the preliminary draft instrument, which had been developed based on the preliminary conceptual framework (Fig. 1; Fig. 2a). Caregivers reported that caring for their child impacted a range of domains, including personal and family relationships, personal time, daily activities, physical and mental health, social life, leisure activities, work productivity, and finances. For example, over half of the participants (9/16; 56%) reported a negative impact on their spousal relationship or time available to spend with their spouse, although some also reported that their spousal relationship had become stronger or closer. All caregivers reported an impact on their ability to participate in social activities (16/16; 100%), and most felt they could not give their other family members as much attention as they would like (15/16; 94%). All caregivers (16/16; 100%) also described how their emotions had changed from the time of their child’s diagnosis to the present time, with some describing going through a grieving process, as though they had already lost their child. Some caregivers also reported experiencing anger (5/16; 31%), to varying extents.

Most caregivers said they had no personal time (11/16; 69%). Almost all described feeling exhausted, tired or fatigued (14/16; 88%), and sleep deprivation was also common (14/16; 88%). In addition, most reported experiencing financial strain because of their child’s illness (13/16; 81%), and half (8/16; 50%) reported being unable to work because of caregiving responsibilities. A similar pattern of saturation was evident for caregivers of children with MLD from the late-infantile and juvenile onset groups. The main impact concepts at the broader domain level (e.g. “participating in social activities”, “feeling worried”, “feeling socially isolated”) were discussed early in the caregiver interviews and were experienced by the majority of caregivers of children with either MLD subtype. In successive interviews, more nuanced and diverse impact concepts from individual caregivers were captured and documented (e.g. “difficulty carrying child”, “dealing with educational issues/school districts”, “no time to shower”). These concepts often provided more context to the main impact, but may not have reached saturation.

#### Stage 1: cognitive debriefing in MLD

The 25-item preliminary draft MLD-CIQ was revised in response to the three rounds of cognitive interviews and translatability assessments. Nine new items were added (Table 3). Wording was modified for 25 items. Examples of wording changes based on translatability assessments included using “patient” instead of “child” and consistently referring to the patient’s “illness” rather than using other terms such as “condition”. Several caregivers also suggested that the word ‘burden’ should be removed from the questionnaire title, expressing that they did not view their child as a burden. This resulted in a change from the caregiver ‘burden’ questionnaire to the caregiver ‘impact’ questionnaire. Items resulting from the stage 1 interviews measured how often a situation was experienced by the caregiver (“never” to “always”), except for the financial question, which measured how much financial strain was experienced (“none” to “a lot”).

The preliminary draft MLD-CIQ utilized a 4-week recall period based on existing caregiver burden instruments [23–26]. After further investigator discussion, the 4-week period was retained for the financial domain only. For the other domains, 4 weeks was considered too long for accurate recall, and this was reduced to 2 weeks. Throughout the MLD interviews, caregivers generally accepted the 2-week recall period. The translation assessment after round 2 identified that the definition of when a week starts and ends can vary; therefore, the description was changed from 2 weeks to 14 days. Following additional consideration of best practice guidelines [16], the recall period was reduced further to 7 days, with the aim of minimizing recall bias while retaining a sufficiently long period to capture relevant experiences. In addition, further to feedback from several caregivers that financial strains are often a long-term issue, the financial domain recall period was set at 3 months.

Review of the CIQ by MLD patient stakeholders did not result in any new concepts being raised and they confirmed the contents of the instrument.

#### Stage 2: concept elicitation in MPS II

The conceptual model developed for MLD was largely confirmed by caregivers of patients with MPS II. One overarching concept raised in the MPS II interviews, not captured in the core CIQ module, was the impact of behavioral symptoms. During concept elicitation, many caregivers (10/14; 71%) described behavioral symptoms, including aggressive behavior, such as kicking and hitting (9/14; 64%); loud vocalizations, or yelling out in frustration or joy (6/14; 43%); and the inability to sit or stand still (3/14; 21%). Other specific behaviors reported by caregivers included being very talkative, being very vocal but using only syllables, growling, grunting, biting others, chewing on things, pulling at arms, stepping on toes, and seeking constant attention. Resulting impacts described by caregivers included the need to constantly supervise their child and the inability to leave them unattended (14/14; 100%), a lack of time to participate in social and leisure activities (13/14; 93%), and difficulty completing daily activities, such as household chores (10/14; 71%).

#### Stage 2: cognitive debriefing in MPS II

The instructions, items, and response options were generally well understood by most participants; only a few minor modifications to wording were needed for clarity. Caregivers generally endorsed the relevance of the items. However, some concepts were found not to be captured (e.g. behavioral symptoms), and two items were added to assess frustration with the patient’s lack of ability to communicate and physical exhaustion owing to the physical requirements of caring for their child. Some items were not considered conceptually essential to incorporate in the instrument, including feeling discouraged about the limited treatment options for the patient’s illness (because treatment may change over the next few years) and feeling worried about the patient (deleted from core domain owing to lack of attribution of the item, e.g. worried about what specifically?) (Table 3). Seven of the eight MPS II caregivers demonstrated clear understanding of the 7-day recall period, so no changes were recommended.

#### Stage 3: cognitive debriefing in MPS IIIA

Caregivers readily endorsed most items presented in round 1 and all items tested in round 2 of the cognitive debriefing interviews as relevant and important in assessing the impact of caring for a patient with MPS IIIA. During round 1, caregivers perceived three items related to concepts in the emotional/psychological functioning domain as important but missing from the CIQ (Table 3). One item (asking about caregiver difficulty dealing with disruptive behavior) was deemed redundant because of another item relating to emotional difficulty and was removed from the CIQ before round 2.

Minor global edits were made to items and response options before round 2 to make the wording more neutral and improve consistency in interpretation, respectively. The word “disruptive” when referring to the patient’s behavior was removed from six items, and the “somewhat difficult” response option was changed to “moderately difficult”. Most items asked about the frequency of occurrence (“never” to “always”), although some (11/30) measured the level of difficulty experienced (“not at all difficult” to “unable to do”).

Following round 2, minor edits were made to several items to make the wording more neutral and improve consistency in interpretation. The phrase “negatively affected” was changed to “impacted” for two items, and “taking care of the patient” was changed to “taking care of the patient’s illness” for two items, for consistency with other modifications. Across both rounds, all participants deemed the instructions and items clear and easy to understand.

During the MPS IIIA interviews, participants in both rounds were able to respond to all items using the 7-day recall period. However, half of the participants recommended a longer recall period than 7 days for the finance-related items: 1 month would reflect the monthly cycle of bill payments. Thus, the recall period for the financial items was changed to “the past month”.

#### CIQ instrument

After completion of all interviews, a revised conceptual framework was generated (Fig. 2b), and the content of the final CIQ was confirmed. The final version has 30 items in five domains: (1) social functioning (7 items); (2) impact on daily activities (5 items); (3) emotional/psychological functioning (10 items); (4) physical functioning (6 items); and (5) financial impact (2 items).

## Discussion

This article documents the sequential development of the CIQ, designed for collecting data about the impacts of caring on caregivers of children with the rare pediatric LSDs MLD, MPS II and MPS IIIA. At each stage of its development, the instrument was endorsed as capturing the relevant and important concepts by caregivers of children with each of the three conditions. Caring for a child or adolescent with an LSD impacts the daily living and social, emotional, psychological, and physical health of caregivers. Social functioning impacts discussed included difficulty in spending time with family and friends and participating in social or leisure activities due to caring responsibilities, as well as feeling isolated from others. The following impacts were reported in all three diseases: feeling helpless because of the patient’s illness and feelings about being unable to communicate with the patient (emotional/psychological); difficulty in making/keeping plans and in completing daily chores/tasks at home or work (daily activities); feeling physically exhausted as a result of caring for the patient, sleep disturbance and inability to take care of own health (physical health); and financial difficulties caused by caring for the patient.

At stage 2, several behavior-related items were added for MPS II that were not captured for MLD. These may be disease-specific, although this possibility has not yet been explored further because the final CIQ instrument was not tested with these disease populations. The emotional/psychological functioning items added for MPS IIIA in stage 3 are likely to be important for all three conditions, given that emotional and psychological impacts were also mentioned by MLD and MPS II caregivers. However, the concepts were not captured by specific items.

The comparison of the preliminary and final versions of the conceptual framework (Fig. 2) demonstrates that although some changes were made based on caregivers’ input, the overall structure and content of the framework remained mostly unchanged. The only domain added was ‘daily activities’, and the majority of changes were to individual items or the wording. This suggests that caring for children with a range of chronic medical conditions impacts upon similar domains of caregivers’ lives, but that there may be disease-specific differences in more nuanced concepts and wording preferences.

Developing an instrument in pediatric rare disease populations can be challenging, owing to factors such as small sample size, difficulty interviewing caregivers of recently diagnosed, early-onset patients, and rapid disease progression. An advantage of the sequential approach used here is that the number of caregivers giving their input is increased, and this can generate a single instrument applicable to multiple diseases. This also offers the benefit of being able to directly compare data across different conditions in which there are overlapping concepts. In contrast, validating and adjusting an existing tool developed for other rare conditions can lead to multiple versions of an instrument that measure similar concepts, thereby limiting the ability to perform direct comparisons and possibly increasing costs. Estimation of the number of participants needed in qualitative research is based on projections of the number of participants needed to reach saturation [20]. Given the practical constraints, developing an instrument in this way may be an effective approach for rare diseases with similar pathology.

We identified 23 existing instruments related to caregiver burden during stage 0 of the study. Although these were used to inform the preliminary draft MLD-CIQ, they had been developed for a range of different contexts and conditions. In many cases, these were produced without direct caregiver feedback, and were not specific to the target disease population of rare pediatric LSDs with their severe motor and cognitive dysfunction. For example, the Child and Adolescent Burden Assessment (CABA) covers several physical, emotional, social and economic impacts [27], but is designed for caregivers of children with psychiatric disorders and therefore may not capture the effects of LSD-specific motor symptoms on caregivers. Similarly, although the Family Impact Module of the generic Pediatric Quality of Life Inventory™ (PedsQL™) [23] could be considered applicable to a wider range of diseases than some of the other instruments, it does not include any financial impacts or disease-specific impacts related to challenging behavior and severe cognitive impairment. Therefore, this may also be unsuitable for capturing the full range of concepts identified among caregivers of patients with LSDs. In addition, even though there are some existing instruments developed for patients with LSDs, these do not focus specifically on caregiver impacts. For example, the Hunter Syndrome-Functional Outcomes for Clinical Understanding Scale (HS-FOCUS), although specific to MPS II and completed by caregivers, focuses on measuring critical functions of patients, such as walking/standing [28]. Moreover, Eichler et al previously surveyed the MLD caregiver perspective to identify relevant clinical/quality-of-life outcomes [14]; however, there were still no available instruments with documented development and validation that capture all the known concepts relevant to MLD.

Next steps in the development of the CIQ will include additional testing in the target samples to confirm item selection/reduction, examination of the conceptual structure of the instrument, and determination of the optimal scoring approach and score interpretation guidelines. Psychometric analysis will be conducted to examine measurement properties such as reliability and validity. The use of electronic formats (e.g. handheld devices) may be explored, and online communities could facilitate the collection of data that are particularly relevant to rare diseases or hard-to-reach populations. For example, previous studies have used the PatientsLikeMe online network to recruit large numbers of participants for the development and completion of surveys relating to chronic health conditions [29, 30].

One limitation of this study is that it only focuses on three LSDs, for which treatments are undergoing clinical trials. It would be useful for additional research to explore the applicability of the instrument to caregivers of patients with other lysosomal or similar pediatric neurodegenerative diseases. A potential limitation of the interviews being conducted mostly by telephone is that non-verbal cues (e.g. body language) from the interviewee could not be observed, possibly affecting interview quality. Furthermore, some developmental stages were conducted by different teams, and although similar processes were followed during each stage, there were some differences. For example, there was no formal concept elicitation during stage 3, although caregivers for patients with MPS IIIA were given the opportunity to mention any concepts they thought were missing, and feedback was not obtained from patient stakeholders for MPS IIIA. A limitation of the sequential approach to instrument development used here is that the final questionnaire (which included minor changes after the interviews with caregivers of patients with MPS IIIA) was not retested by cognitive debriefing with the caregivers of patients with MLD or MPS II. Additionally, in concept elicitation interviews, caregivers were asked to focus on current burden to limit recall bias, which may lead to the exclusion of concepts that are relevant for earlier or later stages of the disease. For example, behavior-related items that are relevant for caregivers of patients with MPS II may not be relevant for caregivers of patients with late-infantile MLD. The population, disease state, and application of the instrument can affect the appropriateness of the recall period. In general, items with short recall periods are usually preferable to decrease recall bias [16].

## Conclusions

These qualitative findings provide support for the relevance of the CIQ for use with caregivers of children with MLD, MPS II or MPS IIIA. Given the challenges of developing an instrument for use with rare diseases, a sequential approach was used, which increased the number of caregivers providing input and generated an instrument with broad applicability. We have identified domains and concepts that may be relevant across these three diseases, and potentially other rare or neurological diseases. However, additional studies, including further psychometric testing with caregivers in the specific diseases, will be needed to confirm the conceptual framework and to finalize the instrument. Nonetheless, this CIQ provides a useful starting point to assess the impact of caring for children with rare LSDs, as well as to compare impacts across different groups of caregivers.

## Additional file

Additional file 1: Interview guide themes. (PDF 95 kb)

## Data Availability

The full interview guides used in CIQ development and interview transcripts are available from the corresponding author and with permission of Shire (a member of the Takeda group of companies) on reasonable request.

## Abbreviations

CIQ: Caregiver Impact Questionnaire
HS-FOCUS: Hunter Syndrome-Functional Outcomes for Clinical Understanding Scale
LSD: Lysosomal storage disease
MLD: Metachromatic leukodystrophy
MPS II: Mucopolysaccharidosis type II
MPS IIIA: Mucopolysaccharidosis type IIIA
MPS: Mucopolysaccharidosis
