# Supplementary material for: Assessing the impact on caregivers caring for patients with rare pediatric lysosomal storage diseases: development of the Caregiver Impact Questionnaire
Source: J Patient Rep Outcomes. 2019 Jul 23;3:44. doi: 10.1186/s41687-019-0140-3 (PMC6650510; doi:10.1186/s41687-019-0140-3)
Supplement: Supplementary file 1 — Interview guide themes. (PDF 95 kb) [file 41687_2019_140_MOESM1_ESM.pdf]

Additional File 1 Interview guide themes

Concept elicitation (MLD and MPS II)

**Section/theme**	**Example questions**
Diagnosis and symptoms	What symptoms did you observe that led you to seek medical care for your child?
	When was your child first diagnosed with [MLD/MPS II]?
	Did you experience any difficulty obtaining a diagnosis?
	How often does your child experience [insert name of symptom]?
	How does treatment or care help your child’s symptoms?
	Is there anyone else who shares caregiving responsibilities with you or assists you in caring for your child with [MLD/MPS II]?
Family impacts	How does caring for your child affect your relationship with your spouse?
	How does caring for your child affect your relationship with your other children?
	What about your ability to pay attention to family members?
Impacts on personal time	How has caring for your child affected your personal time for yourself?
	How does caring for your child affect your daily schedule?
	Do you find that you make plans but then have to cancel or change them?
	How much time do you spend traveling to medical appointments?
Emotional impacts	How does caring for your child affect you emotionally or psychologically?
	What other kinds of emotional impacts do you experience as a caregiver of someone with [MLD/MPS II]?
Social impacts	Do you worry about going out in public with your child?
	How does caring for your child affect your leisure activities outside of the home?
	How does caring for your child affect your interactions with friends?
Physical health impacts	What kinds of physical impacts do you experience as a caregiver of someone with [MLD/MPS II]?
	How does caring for your child affect you physically?
Work and financial impacts	How has caring for your child affected your work or career outside of the home?
	How has caring for your child affected you financially?
	What are the specific costs associated with caring for your child?
	Have you had to reduce your hours or had to change job(s)?
Additional impacts	Are there any issues about how caring for a person with [MLD/MPS II] impacts your life, that we left out, that are really important to ask about?
	Is there anything else you would like to say?

MLD Metachromatic leukodystrophy, *MPS II* Mucopolysaccharidosis II

Cognitive debriefing (MLD, MPS II and MPS IIIA)

Section/theme	Example questions
Understanding of instructions	Did you have any difficulty in understanding these instructions?
	In your own words, what are the instructions asking you to do?
	Are there any words or phrases that you would change to improve the instructions or make them clearer to you?
Phrasing of questions	In your own words, what do you think this question is asking?
	What does the phrase/term/word "[key word/phrase]" mean to you?
	Would you use another phrase to describe "[key word/phrase]"? If yes, what other words would you use?
	How, if at all, could the wording of this question be improved to make it clearer?
Relevance of questions	Was this item relevant to you?
	How relevant is this question to what you experience with caring for your child?
Answering of questions	Why did you choose [response option that was selected]?
	Was this response option difficult or easy to understand?
	What do you think of the response options? Are they different or distinct from each other? Do we have too many? Too few?
Recall period	What timeframe were you thinking about when answering this question?
	Was it difficult or easy to think back over 14 days to come up with your answer? Was the time period too short? Too long? (MLD, third interview round)
	Did you have any difficulty answering any of the items with the 7-day recall period? (MPS II interview)
	What does "the past 7 days" mean to you? What techniques do you use to help you remember this information? (MPS IIIA interview)
Overall thoughts on questionnaire	Have we missed anything important that you think we should add to this questionnaire?
	Which of the questions do you feel is the most important to ask about the impact of caring for a child with [MLD/MPS II/MPS IIIA]?
	Did you feel any of the questions were not needed? If yes, which ones? Why?
	Are there any questions you think are redundant? If so, which ones?
	How easy or difficult was it to complete this questionnaire?
	What suggestions do you have for changing the questionnaire so it is easier to complete?
	Do you have any additional feedback on this questionnaire?

MLD Metachromatic leukodystrophy, *MPS II* Mucopolysaccharidosis II, *MPS IIIA* Mucopolysaccharidosis IIIA
